# Experimental Investigation of the Magnetorheological Behavior of PDMS Elastomer Reinforced with Iron Micro/Nanoparticles

**DOI:** 10.3390/polym9120696

**Published:** 2017-12-10

**Authors:** Luis Manuel Palacios-Pineda, Imperio Anel Perales-Martinez, Luis M. Lozano-Sanchez, Oscar Martínez-Romero, Jesús Puente-Córdova, Emmanuel Segura-Cárdenas, Alex Elías-Zúñiga

**Affiliations:** 1 Tecnologico de Monterrey, Escuela de Ingeniería y Ciencias, Ave. Eugenio Garza Sada 2501, Monterrey 64849, Mexico; palacios@itpachuca.edu.mx (L.M.P.-P.); lmarcelo.lozano@gmail.com (L.M.L.-S.); oscar.martinez@itesm.mx (Q.M.-R.); esca@itesm.mx (E.S.-C.); aelias@itesm.mx (A.E.-Z.); 2 División de Estudios de Posgrado e Investigación, Tecnológico Nacional de Mexico, Instituto Tecnológico de Pachuca, Carr. México-Pachuca km 87.5, Col. Venta Prieta, C.P., Pachuca 42080, Mexico; 3 Facultad de Ingeniería Mecánica y Eléctrica, Universidad Autónoma de Nuevo León, Av. Universidad s/n, Ciudad Universitaria, C.P., San Nicolas de los Garza 66451, Mexico; jesus_ime@hotmail.com

**Keywords:** magnetorheological elastomer, iron micro- and nanoparticles, magnetic and rheological properties, swelling crosslink density, Mullins’ effect

## Abstract

The aim of this article focuses on identifying how the addition of iron micro- and nanoparticles influences the physical properties of magnetorheological composite materials developed with a polydimethylsiloxane (PDMS) matrix with different contents of silicone oil used as additive. A number of characterization techniques have been performed in order to fully characterize the samples, such as cyclic and uniaxial extension, rheology, swelling, Vibrating sample magnetometer (VSM), X-ray Diffraction (XRD), Scanning electron microscopy (SEM), Fourier-Transform Infrared (FTIR), X-ray photoelectronic spectroscopy (XPS) and Thermogravimetric analysis (TGA). The comparison between two matrices with different shore hardnesses and their mechanical and chemical properties are elucidated by swelling and tensile tests. In fact, swelling tests showed that higher crosslink density leads to increasing elongation at break and tensile strength values for the composite materials. The best mechanical performance in the magnetorheological material was observed for those samples manufactured using a higher silicone oil content in a hard polymeric matrix. Furthermore, it has been found that the magnetic properties are enhanced when nanoparticles are used as fillers instead of microparticles.

## 1. Introduction

Engineering elastomers are a group of polymers with high elasticity that are widely used as adaptive dampers in vehicles, gaskets, artificial muscles and actuators, among others. One of the key properties of elastomers is their hardness, which can be adjusted through the choice of the material and the degree of chemical crosslinking. Hardness and density of polymeric materials can influence the overall performance and their use in a specific application.

Magnetorheological (MR) materials form a class of smart materials in which their mechanical properties can be constantly and reversibly adjusted via an external magnetic field [1,2]. MR materials are classified mainly as MR fluids and MR elastomers. In MR fluids (MRF), magnetic particles are suspended into a liquid carrier fluid, while MR elastomers (MRE) consist of an elastomer matrix and magnetizable particles [3,4]. The main advantage of MRE compared to MRF is that particles do not settle with time, owing to the fact that they are locked in the matrix during the curing process [5,6]. MREs are based on silicone rubber, carbonyl iron particles (CIPs) and silicone oil [7,8,9,10]. CIPs are commonly used because of their high magnetization value (up to 2.1 Tesla), high magnetic permeability, soft magnetic characteristics, low residual magnetization and universal availability [11,12]. Almost all research done up until now regarding MREs focused on studying the influence that CIPs have on the physical behavior of the rubber matrix. Commonly, the size of CIPs added into the elastomer matrix is in the range from 1 to 11 µm [10,13,14,15,16,17,18,19,20]. Several authors have studied the effect that magnetized nanoparticles have in magnetorheological suspensions [21,22,23,24,25], as well as in MREs in which nanoparticles of COFe_2_O_4_ [26], SiC [27], Ni [28], FeCo_3_ [29] and Fe [30] were used to develop the composite materials.

Since the mechanical properties of reinforced elastomers depend notably on the particle size and the particle-matrix interface adhesion [31], and since the percentage of particle loading requires further investigation, in one of our previous works [32] the mechanical properties of a MRE composite material, based on a polydimethylsiloxane (PDMS) matrix and different contents (0–40 wt %) of CIPs (2.5 µm), was analyzed. It was found that the appropriate content of magnetic particles added to the polymeric matrix was 20 wt % of carbonyl iron particles. This magnetorheological material showed the best mechanical performance. Taking as reference these findings and also to understand how iron nanoparticles influence the mechanical and magnetorheological properties of the resulting composite materials, in this research work the behavior of the magnetorheological elastomer is compared when nano- and microparticles (20 wt %) are used to reinforce the polymeric matrix. Therefore, the aim of this work focused on studying how the addition of iron magnetic nano- or carbonyl iron microparticles influence the physical, chemical and mechanical properties of polydimethylsiloxane (PDMS) reinforced polymeric materials for different shore hardnesses. It also investigated the influence that the additive volume of silicone oil has on the composite developed materials.

## 2. Materials and Methods

### 2.1. Materials

The materials used to manufacture were dimethyl hydroxy-polydimethylsiloxane (PDMS) P-85 RTV and PE-21 RTV (with shore A hardness of 14 and 20, respectively), polydimethylsiloxane with viscosity of 340 cps used as dispersant agent (silicone oil, SO), and tetraethyl orthosilicate and tin dibutyl laurate as curing agent, all acquired from Polisil (México City, Mexico). Two iron-based magnetic fillers with different particle sizes were used, 70 nm and 2.5 µm of average size, both purchased from Sigma-Aldrich (Monterrey, México).

### 2.2. Fabrication of Isotropic MREs

Firstly, the magnetic particles (20 wt %) and SO were mixed for 3 to 5 min. Then, 44 g of PDMS was added to this mixture and all components were stirred at room temperature for ~5 min. Then, curing agent was added before pouring the homogeneous mixture into a mold. The curing process took place under vacuum conditions at room temperature for 12 h. Contents of 4% and 24% of SO were used based on the PDMS volume to obtain enhanced mechanical properties of MRE materials. The different types of developed materials are listed in Table 1.

### 2.3. Characterization

**Figure 1:**
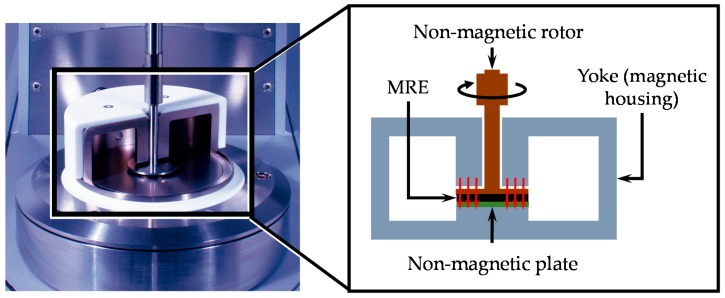
Diagram of the magnetorheological measuring cell. The MRE sample is placed between the bottom plate and the rotor, both made of a non-magnetic material.

The uniaxial strength values of MRE samples were obtained in an universal testing machine Instron 3365 (Instron, Norwood, MA, USA), based on the standard ISO37-2011. The storage modulus (G’) and the loss modulus (G’’) were recorded by using a rheometer Anton Paar, model: MCR301 (Anton Paar GmbH, Graz, Austria). A parallel-plate rotor was installed in the rheometer. Each sample was subjected to a shear-mode oscillatory motion. An electromagnet was used to generate a magnetic flux density of up to 1 T by tuning the DC power supply from 0 to 5 A, as illustrated in Figure 1. All experimental tests were performed at room temperature. To obtain G’ and G’’, cylindrical specimens of 10 mm of diameter with 1 mm thickness were manufactured. By using a Quantum Design Dynacool-I PPMS (Quantum Design, Inc., San Diego, CA, USA) platform and a vibrating sample magnetometer (VSM), the magnetic sample properties were recorded. The magnetic particles and MRE samples were weighed in an electronic balance. Then, each sample was mounted on the top of the vibrating probe kept between two magnetic poles and continuously vibrated mechanically during the analysis. The applied magnetic field was gradually increased from 0 to 1591 kA/m, then it was reduced to −1591 kA/m, and again increased to 1591 kA/m to complete the hysteresis loop. All these measurements were performed at room temperature.

The characterization of the crystalline structure of the iron magnetic particles was performed in a Panalitycal diffractometer (Malvern Panalytical B.V., Eindhoven, The Netherlands) with Cu Kα radiation (λ = 1.54 Å). The particle size distribution of micro- and nanoparticles, the microstructure of isotropic MRE samples, as well as the elemental composition analyses, were all performed by energy dispersive X-ray spectroscopy (EDS) in a Field Emission SEM (FE-SEM, Helios Double Beam 600 high resolution, Thermo Fisher Scientific, Waltham, MA, USA), operated at 5 kV. To observe the magnetic particles, a very small amount of microparticle powder was deposited on carbon tape, while for nanoparticles, a very small amount of nanoparticle powder was put into a vial with anhydrous isopropanol and was kept under stirring in an ultrasonic bath for about 5 to 10 min. Then, by using a syringe, 2 to 3 drops of colloidal mixture were deposited on a copper grid. The sample was analyzed after all the alcohol was evaporated. To obtain the particle size distribution, SEM images with about 300 particles were considered to estimate the particle mean size distribution by DigitalMicrograph software (Gatan Inc., Pleasanton, CA, USA). The dispersion of magnetic particles into the elastomeric matrix was confirmed from MRE flat samples. Furthermore, the EDS characterization technique was considered to identify the elemental chemical composition using a transversal composite material sample.

To determine the possible interaction between iron particles and SO, infrared (IR) spectroscopy analysis was carried out in a Frontier FTIR spectrometer (PerkinElmer, Madrid, Spain). The surface composition was analyzed by X-ray photo-electron spectroscopy equipment (XPS, Thermo Scientific Escalab 250Xi, Waltham, MA, USA) operated at 12.0 kV and 6 mA.

A SDT Q600 (TA Instruments, New Castle, DE, USA) thermo gravimetric (TGA) apparatus was used to perform thermal experiments in the material samples that were heated from room temperature to 900 °C at 10 °C·min^−1^ under argon atmosphere.

**Figure 2:**
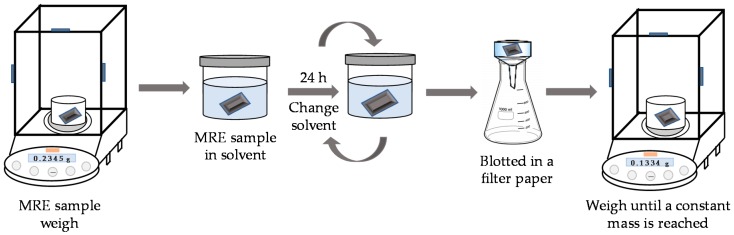
Schematic representation of swelling test procedure.

To assess the influence of reinforced particles in the bare material, a swelling test was performed at room temperature. The samples of dimensions 10 × 7 × 3 mm were weighed and then immersed for 72 h in toluene solvent in a dark environment. Every 24 h the solvent was replaced to minimize interference from toluene-soluble residues in the developed material samples [33]. At the end of the immersion period, the swollen specimens were blotted in a filter paper and then weighed again. Afterwards, specimens were first dried at 80 °C in an oven for a few minutes, and then dried at room temperature until a constant weight was reached. This procedure is illustrated in Figure 2. Each swelling experiment was repeated 3 times for each sample. From the test data, the volume fraction of PDMS was calculated by using the following relationship:

(1) $$Vr=\frac{Vp}{Vp+Vs}=\frac{\frac{m_{dry}}{\rho_{r}}}{\left(\frac{m_{dry}}{\rho_{r}}+\frac{m_{wet}-m_{dry}}{\rho_{s}}\right)},$$

where $m_{wet}$ is the mass of swollen specimen, $m_{dry}$ is the specimen mass until a constant weight was achieved, $\rho_{r}$ is the density of PDMS rubber (1.1 g·mL^−1^), $\rho_{s}$ is the solvent density (for toluene is 0.865 g·mL^−1^). By means of experimental data collected from tension test, the shear modulus (μ) was measured, and by using the Treloar [34] relationship,

(2) $$\mu =\frac{\rho_{r}}{M_{c}}RT=\left[X\right]RT,$$

the crosslinking density value [X] is calculated. In Equation (2), R represents the molar gas constant, T is temperature in *K*, and $M_{c}$ is the mean chain molecular weight between successive points of crosslinkage. Since the crosslink density is related to the polymer volume fraction $V_{r}$ obtained from the swelling test, through the Flory–Rehner equation [35],

(3) [X]=−[ln(1−Vr)+Vr+χVr2]Vo(Vr13−Vr2),

then, the interaction parameter χ between the solvent and the composite material could be determined from this expression.

### 2.4. Material Constitutive Model

To predict the composite MRE mechanical response when material samples are tested under uniaxial loading and unloading cycles, the constitutive material model introduced in [36] is used. From this material model, the Cauchy stress–stretch values could be determined from the following expression.

(4) $$T=\left(1-f\right)ℵB+B\frac{2f}{3}\left(A_{1}+\frac{2A_{2}}{3}\left(I_{1}-3\right)\right)-p1$$

Here, ***B*** and ***T*** are the deformation and Cauchy stress tensors, respectively, *A*_1_ and *A*_2_ are material fitting parameters, *f* describes the percentage of particle volumetric fraction, *p* is a hydrostatic pressure, **1** is the identity tensor, and ℵ is a material response function given by:

(5) $$ℵ=\frac{\mu }{3\lambda_{r}}\left[\beta +\frac{1}{N}\left(\frac{1}{\lambda_{r}}-\frac{1}{\beta \left(1-\lambda_{r}^{2}\frac{2\lambda_{r}}{\beta }\right)}\right)\right],$$

where *N* is the number of links, *µ* is the shear modulus, $\lambda_{r}$ is the relative chain stretch, $\lambda_{r}=\lambda_{chain}/\lambda_{L}$, with $\lambda_{L}=\sqrt{N}$, $\lambda_{\mathrm{chain}}=\sqrt{I_{1}/3}$, *β* is the inverse of the Langevin function given by $\beta ={ℒ}^{-1}\left(\lambda_{r}\right)$, and $I_{1}=\lambda_{1}^{2}+\lambda_{2}^{2}+\lambda_{3}^{2}$ [36,37,38].

Here in this article, we adopt the equation

(6) $$\tau_{j}-\tau_{k}=\{\left[\left(1-f\right)ℵ+\frac{2f}{3}\left(A_{1}+\frac{2A_{2}}{3}\left(I_{1i}-3\right)\right)\right]\left(\lambda_{j}^{2}-\lambda_{k}^{2}\right)\;\;+\frac{G}{2c}\left[\lambda_{j}f_{i}\left(\lambda_{1},\lambda_{2},\lambda_{3}\right)-\lambda_{k}f_{k}\left(\lambda_{1},\lambda_{2},\lambda_{3}\right)\right]\}e^{-b\sqrt{\frac{m}{M}\left(M-m\right)}},\;j\neq k,\;1,\;2,\;3\;\left(no\;sum\right)$$

to describe stress-softened material response behavior [34]. In this Equation (6), *c* represents a constant parameter, *b* is a dimensionless material softening parameter, for uniaxial extension *m* is defined as $m=\sqrt{\lambda^{4}+2\lambda^{-2}}$, and *M* is the maximum strain intensity at the unloading material point.

## 3. Results

### 3.1. Mechanical Properties

**Figure 3:**
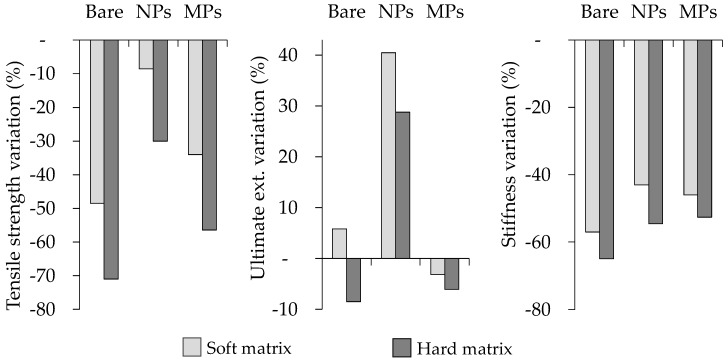
Mechanical properties variation of MRE samples when the SO content is increased from 4% to 24%. SO: silicone oil, NPs: nanoparticles, MPs: microparticles

**Figure 4:**
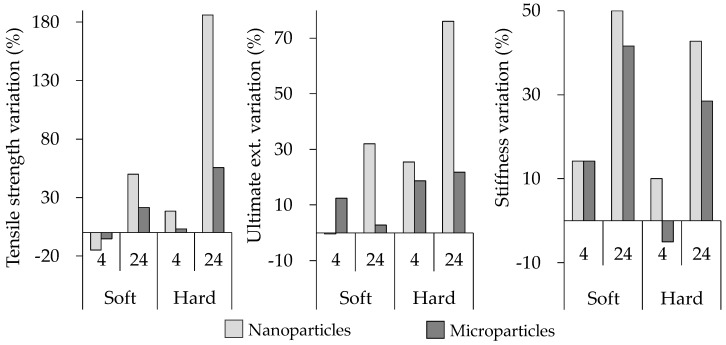
Mechanical properties variation of the reinforced material with micro- and nanoparticles with respect to the bare material.

The mechanical properties in tension of all composite elastomer materials and bare samples are summarized in Table 2. It is observed that samples that contained the soft matrix exhibited higher values of shear modulus (*µ*) and maximum tensile strength (*S_ut_*) compared to the hard-matrix counterparts. The bare sample manufactured with the soft matrix and a low volume of SO (BS04 sample) exhibited the maximum *S_ut_* value of 2.08 MPa. Figure 3 and Figure 4 illustrate the comparison in percentage of the mechanical performance of all MRE samples. The graphs in Figure 3 compare the performance of samples with respect to the SO increase from 4% to 24%. For instance, it is shown that the increase of SO results in a decrease of 49% in *S_ut_* for the bare soft matrix. In fact, it is clearly noted that the increment of SO decreases the *S_ut_* and stiffness values for all samples. Figure 4 compares the performance of all composite materials reinforced with magnetic nano- and microparticles with respect to bare samples. Interestingly, the ultimate extension was enhanced when nanoparticles were used to reinforce the magnetorheological material.

### 3.2. Stress Softening

**Figure 5:**
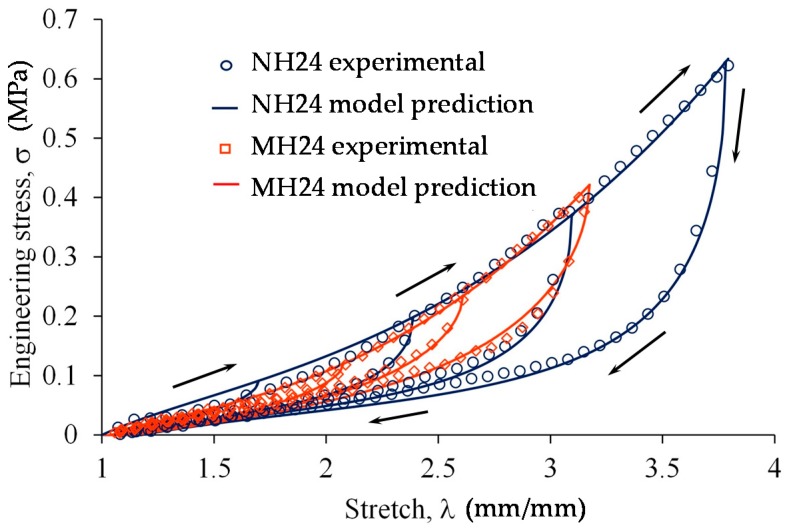
Uniaxial cyclic test for MH24 and NH24 samples, dots are from experimental data and the continuous line is the mathematical model representation. The arrows indicate the direction of load and unload cycle.

A series of cyclic tests in material samples were performed in an Instron universal testing machine. In these tests, the samples reinforced with nano- and microparticles were stretched at the percentage values of 20%, 40%, 60% and 80% of the maximum material sample’s elongation at break. Experimental data curves obtained from the MH24 and NH24 magnetorheological elastomers are shown in Figure 5, in which it is evident that the addition of nanoparticles to the PDMS matrix increases the material energy deformation and the material stiffness when compared to the matrix reinforced with microparticles, as shown in Table 3.

Theoretical predictions to determine the engineering stresses versus the amount of stretch were computed by using Equations (4)–(6). In this case, the experimental data and theoretical predictions are in good agreement (see Figure 5). The material parameters used to plot these curves are summarized in Table 3. For the experimental data plotted in Figure 5 (NH24 and MH24), the estimated number of active links was *N* = 7.6 when nanoparticles were added into the hard PDMS matrix; however, when the material matrix was reinforced with microparticles, the computed value of *N* was 5.4.

### 3.3. Rheological Properties

**Figure 6:**
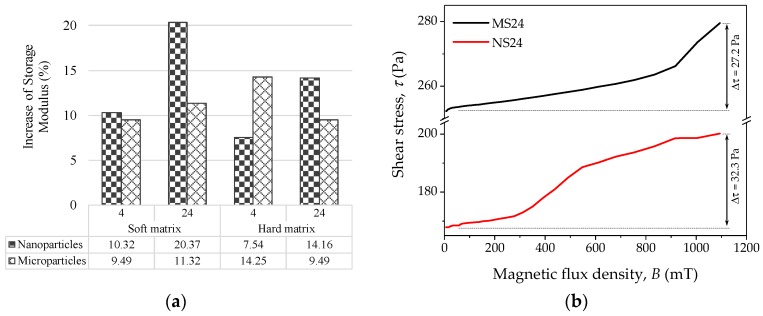
(**a**) Sample increasing storage modulus when a magnetic flux density *B* of 1 T is applied relative to the one measured at the magnetic flux density of 7 mT; (**b**) shear–stress variation for samples MS24 and NS24.

Parallel plate configuration with controlled magnetic field at angular strain of γ=1% and frequency of f = 1 Hz was considered to perform rheological tests on all material samples. The influence of the magnetic flux density (*B*) on the MRE material is summarized in Figure 6a. This experiment was performed varying the magnetic flux density in the range 7 mT ≤ *B* ≤ 1 T. The Figure 6a shows the percentage increase in the storage modulus when a magnetic field of 1 T is applied relative to the one measured at 7 mT. Considering the sample containing the soft matrix with 24% of SO and reinforced with nanoparticles, an increment of 20% in the storage modulus was observed. In all cases, the material samples reinforced with nanoparticles exhibited an enhanced behavior in the magnetic sensitivity when a magnetic field was applied, except in the case of the samples with hard matrix and 4% of SO. In Figure 6b, the increment of shear stress, due to the magnetic flux density, is illustrated for samples MS24 and NS24.

**Figure 7:**
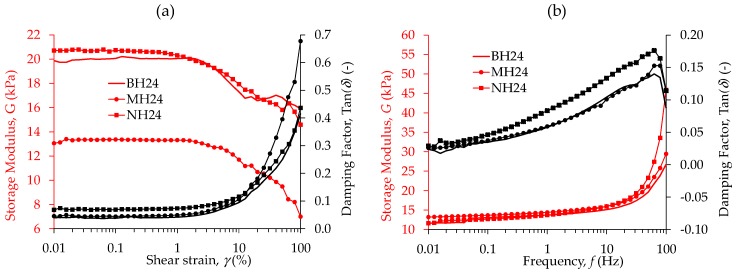
(**a**) Curve of storage modulus *G’* and damping factor tan(δ) obtained from rheological test with controlled shear strain, *f* = 10 Hz and *B* = 0; (**b**) storage modulus *G’* and damping factor tan(δ) obtained from plate-to-plate rheological test with controlled frequency with *γ* = 1% and *B* = 0.

Results from rheological tests are depicted in Figure 7. These data were collected at controlled angular strain (γ), with a frequency of f = 1 Hz and no magnetic field applied. A linear behavior is observed for BH24, MH24 and NH24 samples in the range of 0.01% ≤ γ ≤ 1%. However, there is a significant decrease of about 6 KPa in the G’ of the material sample with microparticles. It is important to mention that, for all samples, the crossover point (tan(δ)=1) is not reached at the shear strain interval values of 0.01% ≤ γ ≤ 100% considered during the measurement tests. It can be seen from Figure 7a that when nanoparticles are used, a slight increment in the storage modulus of 4% is obtained, with an increment in the damping factor of 63% with respect to the bare sample (BH24). The influence of the frequency on G’ and the damping factor is depicted in Figure 7b. The damping factor reaches its maximum value at f = 63 Hz. Here, a constant shear strain of 1% was used in all performed tests.

### 3.4. Magnetic Measurement

**Figure 8:**
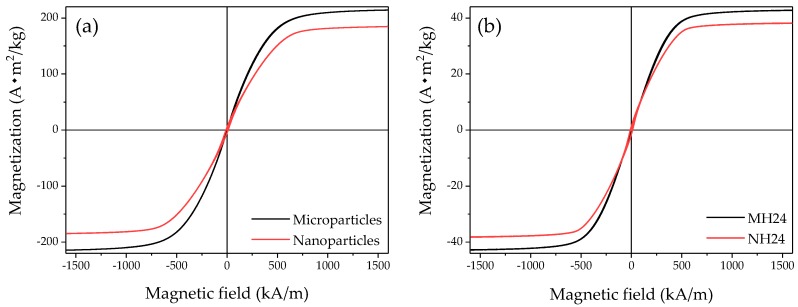
(**a**) Vibrating sample magnetometer VSM data of iron magnetic nano- and microparticle powders; (**b**) hard matrix MRE samples reinforced with nano- and microparticles.

The magnetization curves of micro- and nanoparticle powders are shown in Figure 8. The saturation magnetization obtained at a magnetic field strength of 1591 kA·m^−1^ reached 214 and 184.5 A·m^2^·kg^−1^, for micro- and nanoparticles, respectively. Saturation magnetization values of 43 to 38 A·m^2^·kg^−1^ were measured in PDMS material samples reinforced with micro- and nanoparticles, respectively, as seen in Table 4 and Figure 8b. It is seen from Figure 8 that nanoparticles exhibit a lower magnetization of about 14% compared to microparticles when the magnetic field exceeds 557 kA·m^−1^. A similar trend was reported by Zhang et al. [39]. The magnetic saturation field value between iron particle powder and the composite material drops from ~ 200 to ~ 40 A·m^2^·kg^−1^ because the iron particle content added into the PDMS matrix is only 20 wt %. A marginal change in the saturation magnetization value of the samples manufactured at different SO volumes is evident from the recorded experimental data shown in Table 4. However, note that the saturation value of the composite samples is about one fifth of raw iron nano- and microparticles. It is noteworthy that high-saturation magnetization allows the development of a strong magnetic field, which could enable the design of smaller and lighter components with enhanced magnetorheological performance [40], and to manufacture planar and 3D microstructures via soft lithography [41], since nanoparticles have a lower saturation value with a higher magnetic sensitivity, as illustrated in Figure 8.

### 3.5. Morphological and Structural Analysis

**Figure 9:**
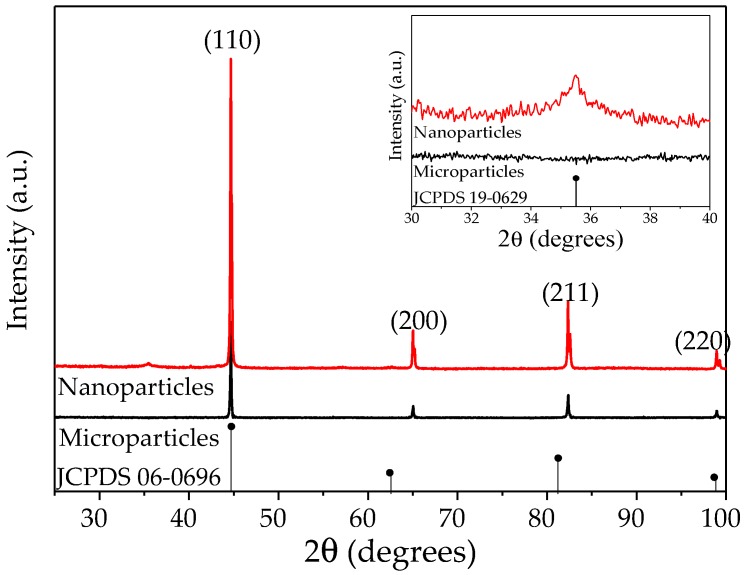
XRD patterns of nano- and microparticle powders.

X-ray diffraction (XRD) measurements were conducted to obtain the patterns of iron nano- and microparticles, as exhibited in Figure 9. It is observed from Figure 9 that both particles have a well-defined crystal structure, which corresponds to α-Fe (JCPDS 06-0696) with the main diffraction peaks at 44.5°, 65° and 82° in 2Ɵ. Additionally, the iron nanoparticle powder exhibited an additional crystallographic structure that corresponds to magnetite, Fe_3_O_4_ (JCPDS 19-0629), which is evident in the inset of Figure 9 with the main diffraction peak at 35.5°.

**Figure 10:**
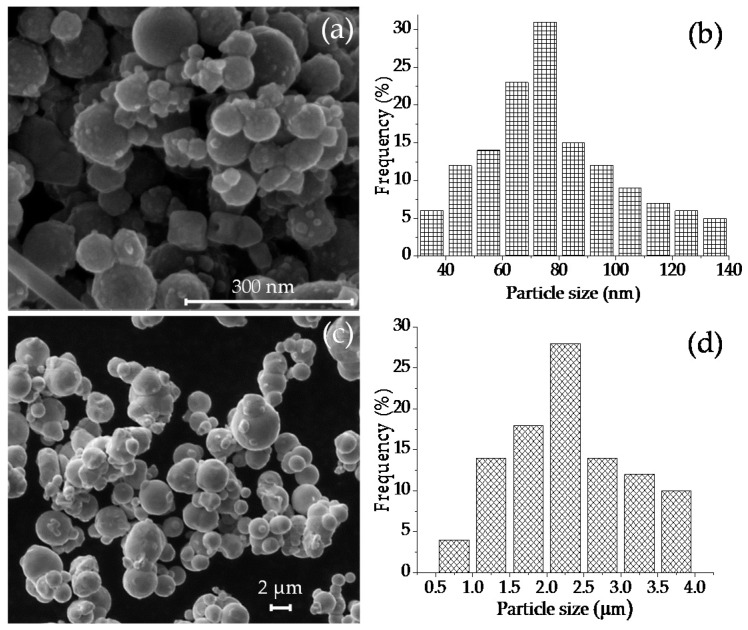
(**a**,**c**) illustrate SEM images of nano- and microparticles, while illustrations (**b**,**d**) exhibit their corresponding particle statistical distribution size.

**Figure 11:**
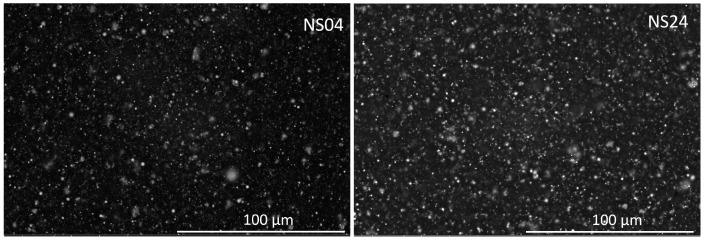
SEM image of NS04 and NS24 samples.

**Figure 12:**
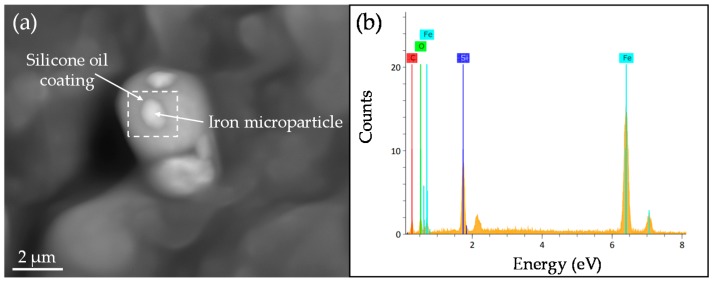
(**a**) SEM image of MS04 sample showing the silicone oil coating, (**b**) Energy dispersive X-ray spectroscopy spectrum analysis of square zone in Figure 12a.

Figure 10 shows SEM images of iron nano- and microparticles. A similar spherical morphology is observed for both kinds of iron particles. The calculated average sizes of particles are shown in the distribution histograms displayed in Figure 10b,d. The measured average sizes were 70 nm and 2.3 µm, for nano- and microparticles, respectively. The dispersion of the nanoparticles by adding low and high contents of SO in a soft matrix is shown in Figure 11. Here, it is observed that an increase in the SO volume does not influence the dispersion of the nanoparticles. Figure 12 shows that the particles are surrounded by a shadow which is due to the silicone oil used to disperse the magnetic particles prior to the curing process. The square represents the region where the energy dispersive spectroscopy analysis was performed. This analysis determined the elemental chemicals of iron, carbon and silicon due to silicone oil that was added into the composite material.

### 3.6. FTIR and XPS Characterization

**Figure 13:**
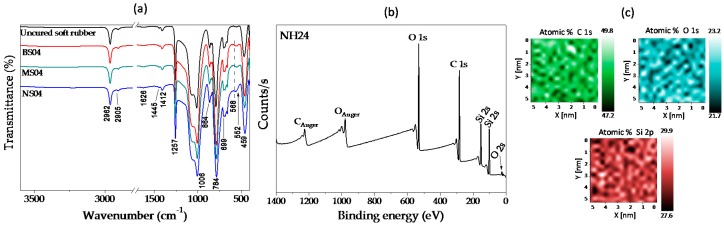
(**a**) FTIR spectra of samples manufactured using a soft matrix with 4% of SO; (**b**) X-ray photoelectronic spectroscopy (XPS) of NH24 sample showing the presence of carbon (C), oxygen (O) and silicon (Si) elements; (**c**) XPS mapping with the elemental composition.

Figure 13 shows the Fourier-transform infrared spectroscopy (FTIR) results of materials based on soft PDMS matrix (shore A = 14). The black curve corresponds to the uncured PDMS, the red one corresponds to bare cured PDMS, while the green and blue curves correspond to the composite materials containing micro- and nanoparticles, respectively. All spectra exhibit the main absorption bands at 2905–2960, 1445, 1415, 1258 and 1005 cm^−1^, which are assigned to the vibration modes −CH_2_− stretching in −Si−CH_2_− [42], C−H bending in −Si−CH_2_− [43], Si−CH= CH_2_ mode [43], symmetric −CH_3_ deformation in −Si−CH_3_ [42,44], and to the Si−O−Si stretching vibration of the crosslinked PDMS, respectively. Furthermore, the band around 875 cm^−1^ is due to the bending motion of Si−OH [45], and the one at 790 cm^−1^ is attributed to −CH_3_ rocking and −Si−C− stretching in −Si−CH_3_ [42,44]. Furthermore, for samples manufactured with iron nanoparticles, we observed a band around 560 cm^−1^ that is characteristic of Fe–O vibrations of iron oxides [46]. That band of Fe−O is due to nanoparticles exhibiting a small amount of oxide, whose crystallographic structure was identified by XRD analysis (inset of Figure 9). Therefore, it can be concluded that there is not a modification in the main functional groups of silicone rubber due to the manufacturing process of the MRE samples. Also, and based on the experimental results discussed above, it is concluded that there is no chemical interaction between the iron particles and the PDMS matrix, since the iron particles are coated by the SO used to disperse the particles prior to the curing process. Similar experimental results were obtained in all the material samples examined with FTIR. To further validate these findings, XPS experimental analysis was performed on the material sample with 24% of SO. Figure 13b shows the peaks that were detected on the surface of the MRE sample manufactured with nanoparticles (NH24). Elemental composition of carbon (C), oxygen (O) and silicon (Si) was identified through the chemical map shown in Figure 13c. Bonds of C−C/C−Si and O−Si in the PDMS matrix [32] were identified by their binding energies with their corresponding peak positions. No iron signals would be expected to appear since this XPS characterization technique is based on a surface analysis and thus, it is not possible to measure beyond 10 nm of the sample surface.

### 3.7. Thermal Stability

**Figure 14:**
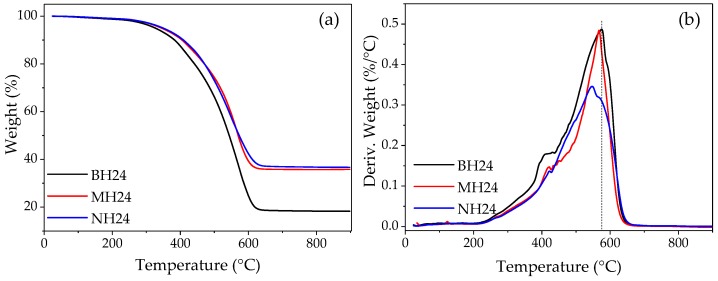
(**a**) TGA thermograms; (**b**) DTGA curves of MRE manufactured using a hard matrix and high SO volume.

Thermogravimetric analysis shows a single-step degradation behavior of the MRE. In fact, Figure 14 shows the TGA and DTGA curves from which the values of T_10%_, Tp, Rp, and the residues of all samples were obtained [46]. These values are listed in Table 4. From this Table 4, it can be seen that there is a gradual decrease in the residues from 25.6% to 21.3% in BS00, BS04 and BS24 samples, when the SO volume is increased from 0% to 24%. On the contrary, the T_10%_ value is increased as SO volume is increased as well. The same tendency is observed for bare samples manufactured with the hard matrix. This behavior could be due to crosslinking reactions occurring at different temperatures. Also, a slight weight increase in the iron nano- and microparticles has been observed mainly due to particle oxidation processes that occurred because of the argon gas impurities.

Based on the TGA and DTGA material sample analysis, the enhancement of the MRE composite material thermal stability is not only due to the addition of iron nano- and microparticles, but also to the increase of the SO content, as shown in Table 4. An interfacial interaction between the PDMS matrix and the iron particles has been enhanced because of the particles’ surface modification with the silicone oil that is acting as coupling agent. Therefore, increasing the SO content led to an enhancement of the interfacial compatibility between the iron particles and the material matrix, resulting in a better thermal stability. Moreover, from the DTGA curves shown in Figure 14b, the composite material experienced a maximum decomposition rate *T_p_* at a lower temperature.

### 3.8. Relationship between Polymer Swelling and Tension Tests

**Figure 15:**
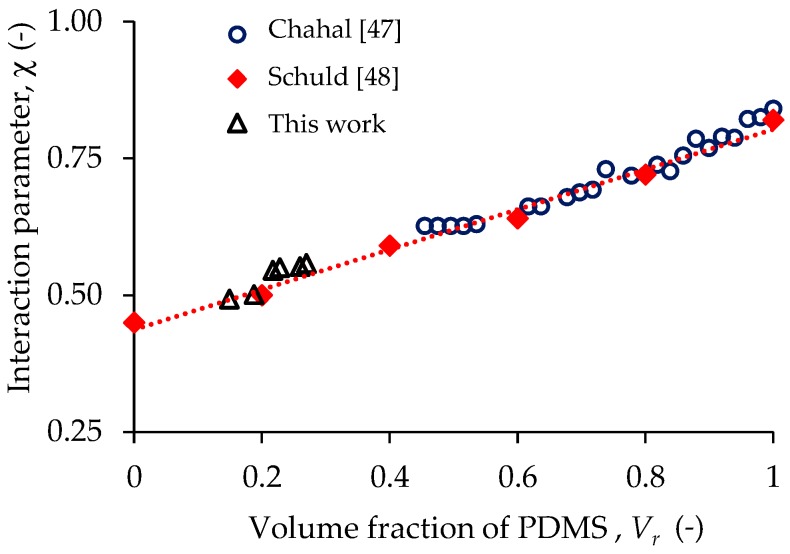
Computed interaction parameter values from Equation (3), compared to those reported by Chahal [47] and by Schuld [48].

The mechanical behavior of PDMS elastomers reinforced with magnetic particles can be explained from the results obtained from swelling and tensile tests. The connection between these tests is established through the interaction parameter χ. Firstly, the volume fraction of PDMS is obtained from the swelling test; secondly, crosslinking density is obtained from the tensile experimental data; and finally, the interaction parameter value is found. In Figure 15, the values between the interaction parameter obtained from Equation (2) are represented by the black triangles. Figure 15 exhibits a good agreement between our computed results and those obtained by Chahal [47] (blue circles) and by Schuld [48] (red diamonds), and it is also clear to see the dependence of the interaction parameter on the polymer volume fraction. Therefore, it is concluded that the swelling and tensile tests aid to measure the crosslinking properties of the developed composited MREs.

**Figure 16:**
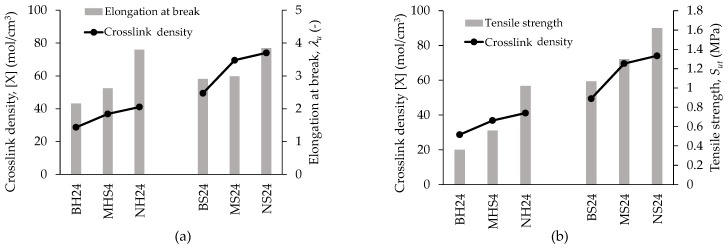
Influence of filler size and matrix hardness on: (**a**) elongation at break, and (**b**) tensile strength.

Figure 16 illustrates that the crosslink density and the tensile strength in general are higher in soft matrix materials because these materials have more chains linked together by covalent bonds, while in the hard matrix there are less crosslinked chains. In conclusion, increasing crosslink density leads to higher tensile strength due to those covalent bonds that keep the polymeric chains together, and therefore, more energy is necessary to break them.

### 3.9. Summary

As a summary, Table 5 shows a comparison of properties between MRE reinforced with nanoparticles and the bare elastomer. The hard matrix with 24% of SO shows the best performance for strength, ultimate extension and thermal stability (10% weight loss, T_10%_), while using a soft matrix with the same amount of SO (24%) exhibited the second-best performance in stiffness properties.

Finally, Table 6 shows a comparison of physical properties recorded experimentally from the composite material samples. It can be concluded from these that when nanoparticles are added into the PDMS matrix, their physical and mechanical properties are higher than those recorded from samples reinforced with microparticles.

## 4. Conclusions

Mechanical, physical and chemical properties were experimentally obtained in several MRE samples reinforced with the addition of 20 wt % of iron micro- and nanoparticles. It has been found that in general, the addition of iron nanoparticles enhances the MRE mechanical and magnetic properties. In fact, the addition of iron nanoparticles as a filler in the hard matrix along with 24% of SO has proved to create the composite material that exhibits the best properties. Chemical interactions between the iron micro- and nanoparticles and the polymeric matrix were not observed from the FTIR and XPS experimental analysis, since these particles are coated by the SO that is used to disperse the particles. TGA experimental results show that this coating enhances the thermal stability. Based on the swelling test results, it has been seen that increasing crosslink density leads to higher tensile strength due to covalent bonds that keep the polymeric chains together, and therefore, more energy is necessary to break them, which was evidenced by tensile tests. Furthermore, it has been found from rheology experimental tests that if iron nanoparticles are added into the silicone matrix, an increment of up to 4% and 63% in the storage modulus and in the damping factor is obtained with respect to the bare samples, respectively. VSM saturation magnetization curves show a slight decrease for the composite materials reinforced with iron nanoparticles, however, their storage modulus increases up to 20% when 1 T of magnetic field is applied. This increment is observed when a soft matrix is reinforced with 20 wt % of iron nanoparticles and 24% of SO. In most of the cases, the material response to a magnetic stimulus improves when nanoparticles are used, except in the case of a hard matrix with 4% of SO. Finally, good dispersion of the magnetic particles into the composite material was observed from SEM analysis, while XRD analysis confirms that both kinds of iron particles have the same crystalline structure. Therefore, moderate magnetic fields could be applied if the composite material with iron nanoparticles possesses the appropriate magnetic saturation. This could enable the manufacturing of lighter and smaller components with enhanced magnetorheological performance.

## Figures and Tables

**Table 1:** Nomenclature definition of magnetorheological elastomer (MRE) samples.

Nomenclature	Filler	Shore A hardness	Silicone oil volume (%)
BS04	Bare	14 (Soft)	4
BS24	Bare	14 (Soft)	24
BH04	Bare	20 (Hard)	4
BH24	Bare	20 (Hard)	24
NS04	Nanoparticles	14 (Soft)	4
NS24	Nanoparticles	14 (Soft)	24
NH04	Nanoparticles	20 (Hard)	4
NH24	Nanoparticles	20 (Hard)	24
MS04	Microparticles	14 (Soft)	4
MS24	Microparticles	14 (Soft)	24
MH04	Microparticles	20 (Hard)	4
MH24	Microparticles	20 (Hard)	24

**Table 2:** Shear modulus *µ*, tensile strength (*S_ut_*) and the elongation at break (*λ_L_*) obtained from uniaxial tensile tests.

Material sample identification	*µ* (MPa)	*S_ut_* (MPa)	*λ_L_* (-)
BS04	0.28	2.08	3.75
BS24	0.12	1.07	3.91
BH04	0.20	1.25	3.35
BH24	0.07	0.36	3.15
NS04	0.32	1.76	3.74
NS24	0.18	1.61	4.85
NH04	0.22	1.48	3.95
NH24	0.10	1.03	4.8
MS04	0.32	1.97	4.09
MS24	0.17	1.30	3.99
MH04	0.19	1.29	3.79
MH24	0.09	0.56	3.62

**Table 3:** Material constants used to fit the cyclic experimental data.

Sample	*μ* (MPa)	*N* (-)	*A*_1_ (MPa)	*A*_2_ (MPa)	*b* (-)	*c* (MPa)	*f* (-)	Permanent set
MS04	0.32	7.5	−2.410	0	0.6	5	0.033	1.11
NS04	0.32	10.0	0.745	0	0.7	7	0.033	1.06
MS24	0.17	6.3	−0.428	0	0.5	10	0.033	1.07
NS24	0.18	5.0	0.645	0	0.6	5	0.033	1.08
MH04	0.19	5.8	−0.473	0	0.5	8	0.033	1.03
NH04	0.22	5.5	−0.481	0	0.65	8	0.033	1.04
MH24	0.09	5.4	−0.622	0	0.4	10	0.033	1.04
NH24	0.1	7.6	−0.048	0	0.6	5	0.033	1.08

**Table 4:** Measured material samples’ TGA and derivative of the thermogravimetric curve (DTG), and the saturation magnetization of MRE (measured by VSM) parameter values.

Sample	T_10%_ (°C)	T_p_ (°C)	R_p_ (%/°C)	Residue (%)	Magnetization (A·m^2^·kg^−1^)
Nanoparticles	N/A	N/A	N/A	102.2	184.7
Microparticles	N/A	N/A	N/A	104.1	214.2
BS00	372.7	574.6	0.437	25.6	N/A
BH00	371.2	585.5	0.484	23.1	N/A
BS04	374.5	577.5	0.466	24.1	N/A
BS24	392.8	579.0	0.556	21.3	N/A
BH04	379.8	583.5	0.483	21.9	N/A
BH24	383.3	575.2	0.487	18.3	N/A
NS04	394.0	552.5	0.292	42.0	38.2
NS24	404.8	571.3	0.315	39.0	39.7
NH04	401.5	564.8	0.324	39.6	39.8
NH24	409.3	547.3	0.346	36.7	38.2
MS04	398.7	556.9	0.402	39.9	42.1
MS24	407.9	563.6	0.464	36.7	42.2
MH04	396.0	568.6	0.409	38.4	42.8
MH24	405.5	567.3	0.484	36.8	42.7

**Table 5:** Influence of nanoparticles on the properties of MRE compared to bare elastomer.

Sample ratio properties	Strength (%)	Elongation at break (%)	Stiffness (%)	Thermal stability (T_10%_) (%)
NS04/BS04	−16	−0.4	14	5
NS24/BS24	50	32	50	3
NH04/BH04	17	26	10	6
NH24/BH24	186	77	42	7

**Table 6:** Influence of nanoparticles on the properties of MRE compared to microparticle-reinforced elastomers.

Sample ratio properties	Strength (%)	Elongation at break (%)	Stiffness (%)	Magnetic field sensitivity (%)	Thermal stability (T_10%_) (%)
NS04/MS04	−11	−11	0	9	−1.2
NS24/MS24	24	29	6	80	−0.8
NH04/MH04	15	6	16	−47	1.4
NH24/MH24	84	45	11	49	0.9
